# Ferroptosis: a promising therapeutic target for periodontitis

**DOI:** 10.3389/fimmu.2026.1823915

**Published:** 2026-04-29

**Authors:** Yuanyuan Li, Libo Sun, Jing Xie, Liumo Li, Ling Guo, Yuyan Lan

**Affiliations:** 1 Oral & Maxillofacial Reconstruction and Regeneration of Luzhou Key Laboratory, The Affiliated Stomatological Hospital, Southwest Medical University, Luzhou, China; 2 Department of Prosthodontics, The Affiliated Stomatological Hospital of Southwest Medical University, Luzhou, Sichuan, China; 3 Institute of Stomatology, Southwest Medical University, Luzhou, China; 4 Department of Stomatology, Luzhou People’s Hospital, Luzhou, China

**Keywords:** ferroptosis, GPx4, lipid peroxidation, oxidative stress, periodontitis, targeted therapy

## Abstract

Periodontitis is a chronic inflammatory disease driven by dysbiotic biofilm and abnormal host adaptive immune responses, characterized by irreversible destruction of periodontal tissues. Recent studies indicate that ferroptosis—a form of iron-dependent lipid peroxidation-mediated cell death—is not a passive outcome of the disease process but rather an important active inducer and pathological amplifier in the development of periodontitis. This review synthesizes existing evidence to propose that ferroptosis acts as an important regulatory node within the pathological network of periodontitis, impairs epithelial barrier function, suppresses osteoblast differentiation and function, and amplifies pro-inflammatory signaling pathways through cell-type-specific mechanisms. Furthermore, ferroptosis exhibits complex cross-regulation with other cell death pathways (e.g., pyroptosis, apoptosis, autophagy), collectively forming a self-amplifying vicious cycle that persistently drives chronic inflammation and tissue destruction. Although iron death-targeting interventions, including iron chelators, GPX4 agonists, lipid peroxidation scavengers, natural bioactive compounds (e.g., curcumin, resveratrol), and nanodelivery systems, show promise in preclinical studies, the cell type specificity and context dependency of ferroptosis pose critical challenges for precise intervention. Therefore, this review positions ferroptosis as a pivotal node in the regulatory network of periodontal cell death, advocating for the development of stage-specific and cell-type-specific targeted strategies. This signifies a paradigm shift in periodontal therapy, transitioning from traditional antimicrobial approaches toward host-centered precision periodontal medicine.

## 1 Introduction

Periodontitis is a globally prevalent chronic inflammatory disease triggered by dysregulation of oral biofilm and host immune responses, serving as the primary cause of adult tooth loss (1). Its global prevalence ranges from 20% to 50%, imposing a significant socioeconomic burden on healthcare systems. When complicated by systemic conditions such as diabetes and cardiovascular disease, it further exacerbates overall health risks (2, 3). The pathological progression of periodontitis is gradual: beginning with gingivitis characterized by gum inflammation and bleeding, it advances to destructive periodontitis marked by irreversible periodontal ligament damage and alveolar bone resorption, ultimately leading to tooth loosening and loss (4, 5). Notably, the chronic inflammatory microenvironment of periodontitis is characterized by persistent pathogen colonization, recurrent bleeding, and continuous release of pro-inflammatory cytokines, creating conditions conducive to dysregulated cell death. The resulting tissue damage far exceeds the direct destruction caused by bacterial infection alone. Factors such as metabolic dysregulation and disruption of the local inflammatory microenvironment significantly influence disease onset, progression rate, and treatment efficacy by modulating iron metabolism, lipid peroxidation, and antioxidant defense systems. This further underscores the necessity of elucidating the molecular mechanisms of ferroptosis and developing targeted therapeutic strategies.

A multi-platform analysis of human gingival crevicular fluid reveals that ferroptosis is an associated regulated cell death mechanism during the clinical progression of periodontitis (6). Ferroptosis is an iron-dependent, lipid peroxidation-driven form of programmed cell death, characterized by disrupted iron homeostasis, excessive accumulation of lipid peroxides, and impaired antioxidant defense systems (7). Clinical studies have confirmed iron overload in periodontitis patients, along with significantly elevated levels of lipid peroxidation products (e.g., 4-hydroxynonenal, malondialdehyde), which correlate positively with disease severity (8). Recent research further reveals that ferroptosis in periodontal ligament cells, gingival epithelial cells, and macrophages directly contributes to periodontal tissue destruction, while the keystone pathogen *Porphyromonas gingivalis* can actively induce ferroptosis through multiple pathways (9). These findings collectively indicate that ferroptosis is deeply involved in the initiation and progression of periodontitis and may serve as an important pathogenic driver.

This review systematically summarizes the molecular mechanisms of ferroptosis in periodontitis, with a focus on how dysregulated iron metabolism, reprogrammed lipid metabolism, and suppressed antioxidant defenses in the periodontal microenvironment regulate ferroptosis. It also explores the cell-type-specific pathological effects of ferroptosis in periodontal cells and its crosstalk with other modes of cell death (e.g., pyroptosis, autophagy, apoptosis). Furthermore, potential therapeutic strategies targeting ferroptosis are evaluated, and the future directions of research on the association between periodontitis and ferroptosis are as follows. This work aims to provide novel insights into the pathogenesis of periodontitis and to establish a theoretical foundation for developing host-directed precision therapies.

## 2 Molecular mechanisms of ferroptosis links to periodontitis

**Figure 1:**
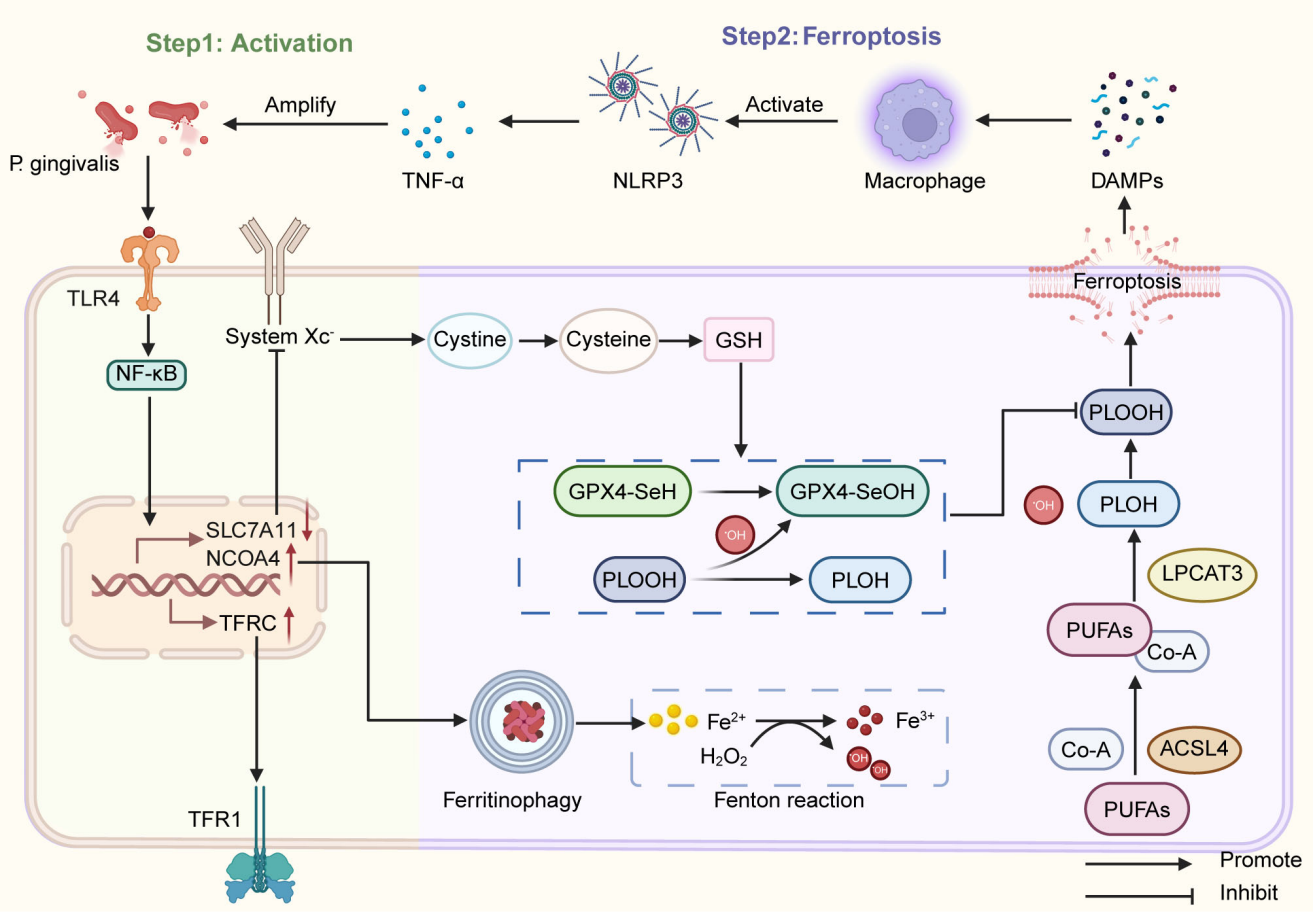
*P. gingivalis*-induced ferroptosis amplifies inflammatory responses. This figure systematically elucidates how *P. gingivalis* synergistically regulates three core pathways via the TLR4/NF-κB signaling pathway: (1) iron metabolism imbalance (upregulation of TFRC and NCOA4 expression leading to intracellular iron overload), (2) lipid metabolism reprogramming (enhanced ACSL4 and LPCAT3 activity promoting polyunsaturated fatty acid phospholipid accumulation), (3) antioxidant defense collapse (downregulation of SLC7A11 and GPX4 triggering glutathione depletion and impaired lipid peroxide clearance). These collectively trigger ferroptosis in periodontal ligament cells and gingival epithelial cells. The damage-associated molecular pattern released by ferroptosis activates the NLRP3 inflammasome in macrophages. Pro-inflammatory factors (e.g., TNF-α) reciprocally enhance ferroptosis susceptibility, forming a self-amplifying vicious cycle that ultimately leads to degradation of periodontal supporting tissues and alveolar bone resorption. Arrows denote activation; T-bars denote inhibition. Key molecular abbreviations are detailed in the main text.

Ferroptosis is a regulated form of cell death driven by iron-dependent lipid peroxidation, characterized by dysregulated iron metabolism, accumulation of lipid peroxides, and impaired antioxidant defense. While its molecular mechanisms have been well-established in fields such as oncology and neurodegenerative diseases, its specific regulatory network and pathological significance in periodontitis remain in the early stages of investigation. Current studies have primarily confirmed the presence of iron overload and elevated lipid peroxidation in periodontitis patients; however, there is a lack of systematic analysis regarding the role of ferroptosis in the chronic progression of the disease. In summary, this section will elucidate the molecular mechanisms of ferroptosis in periodontitis, with a focus on the keystone pathogen *P. gingivalis* as a central inducer. This bacterium drives ferroptosis in periodontal cells by modulating iron metabolism, lipid metabolism, and the antioxidant system, thereby forming a self-amplifying inflammatory circuit that exacerbates disease progression. The main processes involved are illustrated in **Figure 1**.

### 2.1 Dysregulated iron metabolism drives ferroptosis in periodontitis

Iron is an essential trace element for cellular physiological activities, and its homeostasis is tightly regulated by a complex network encompassing iron uptake, storage, release, and efflux. Within the local inflammatory microenvironment of periodontitis, the unique anatomical structure of the periodontal pocket provides ideal conditions for iron metabolism dysregulation. Features such as hypoxia, low pH, and a high concentration of serum components render iron overload substantially more pronounced than in other tissues.

#### 2.1.1 Enhanced iron uptake coupled with imbalanced storage and release

The pathological cascade of iron dysmetabolism in periodontitis is synergistically driven by aberrant iron uptake and pathological iron release, the hypoxic condition of the periodontal pocket plays a key amplifying role in this process. Periodontal pathogens induced by infection and pro-inflammatory cytokines activate the NF-κB signaling pathway (10), upregulating the expression of transferrin receptor 1 (TFRC). Notably, the hypoxic environment of the periodontal pocket further synergistically upregulates TFRC by stabilizing hypoxia-inducible factor-1α (HIF-1α) and enhancing the endocytosis of transferrin-bound ferric iron (Fe^3+^) (11). This results in intracellular iron accumulation and initial impairment of periodontal ligament cell (PDLC) function (12). Concurrently, inflammatory factors and oxidative stress pathologically activate nuclear receptor coactivator 4 (NCOA4)-mediated ferritinophagy. Research by Guo et al. demonstrated that *P. gingivalis* infection in periodontitis upregulates NCOA4 via the NF-κB pathway, promoting ferritin lysosomal degradation and causing a continuous influx of ferrous iron (Fe^2+^) into the labile iron pool (LIP) (13). Furthermore, dysfunction of the heme-centric negative regulatory system of iron metabolism weakens its feedback inhibition on iron accumulation and iron toxicity (14). This further exacerbates iron homeostasis imbalance, directly impairing mitochondrial function in periodontal supporting cells to reduce cellular viability and indirectly promoting periodontal ligament structural destruction (15).

#### 2.1.2 Impaired iron efflux and pathological iron accumulation

Recurrent microbleeding within the periodontal pocket is a core feature distinguishing periodontitis from other chronic inflammatory diseases and represents the most direct source of iron overload. Continuous microtrauma caused by physiological activities such as toothbrushing and mastication renders the gingival crevicular fluid (GCF) a filtrate rich in hemoglobin (16). Under the low-pH conditions of the periodontal pocket, hemoglobin is more readily dissociated from erythrocytes and subsequently efficiently taken up by infiltrating macrophages via the scavenger receptor CD163, followed by degradation via heme oxygenase-1 (HO-1) to release Fe^2+^ (17). Zhao et al. found that HO-1 expression was significantly elevated in the gingival tissues of periodontitis patients and positively correlated with the degree of iron overload (18). Stabilization of HIF-1α in the hypoxic periodontal pocket further upregulates HO-1, accelerating heme degradation and Fe^2+^ release, thereby forming a positive feedback loop of hypoxia-reinforced iron capture.

Concurrently, inflammation-induced impairment of iron efflux exacerbates iron retention. Inflammation upregulates hepcidin, promoting the internalization and degradation of the iron exporter ferroportin (FPN), thereby effectively blocking cellular iron efflux (14, 19). Although compensatory mechanisms such as the prominin2-multivesicular body-exosome-ferritin axis exist, they are insufficient to counteract aberrant iron influx (20), resulting in iron deposition within cells and in gingival connective tissue. This establishes a sustained reactive oxygen species(ROS)-responsive iron reservoir that serves as a substrate for ferroptosis (18).

Clinical studies have confirmed that ferritin levels in GCF are significantly elevated in periodontitis patients and positively correlate with probing depth (PD) and clinical attachment loss (CAL) (21). Ultimately, excess intracellular Fe^2+^ catalyzes the Fenton reaction, generating highly reactive hydroxyl radicals that provide the initial chemical driving force for the chain reaction of lipid peroxidation (22).

### 2.2 Lipid metabolic reprogramming and lethal peroxidation execution

The execution of ferroptosis is fundamentally driven by lethal peroxidation of membrane lipids, a process involving a series of active lipid metabolic reprogramming events. The core of this reprogramming lies in the alteration of membrane phospholipid composition, particularly an increased proportion of polyunsaturated fatty acids (PUFAs), thereby enhancing membrane lipid susceptibility to oxidative stress.

#### 2.2.1 Regulatory mechanisms of membrane phospholipid remodeling

Lipid metabolic reprogramming in periodontitis is driven by the ACSL4/LPCAT3 axis. Acyl-CoA synthetase long-chain family member 4 (ACSL4) catalyzes the activation of PUFAs, while lysophosphatidylcholine acyltransferase 3 (LPCAT3) precisely incorporates them into the sn-2 position of membrane phospholipids (e.g., phosphatidylethanolamine). This enriches the membrane with oxidation-sensitive PUFA-containing phospholipids (PUFA-PLs) (23). The upregulation of this axis is considered a key hallmark of pro-ferroptotic lipid metabolism.

Periodontitis upregulates this axis through multiple periodontium-specific mechanisms. First, *P. gingivalis* lipopolysaccharide activates downstream inflammatory factors and transcriptional networks via the Toll-like receptor 4 (TLR4)/NF-κB pathway, leading to upregulation of the ACSL4/LPCAT3 axis (24). Second, the hypoxic environment of the periodontal pocket directly upregulates expression of the fatty acid transporter CD36 via HIF-1α, increasing cellular uptake of extracellular fatty acids and providing a more abundant substrate for ACSL4 (25, 26). Furthermore, short-chain fatty acids such as butyrate produced by periodontopathic bacterial metabolism may participate in upregulating ACSL4 expression through epigenetic regulation, although the underlying mechanisms remain to be further elucidated (27). Collectively, these three mechanisms render periodontal ligament cells and other cell types substantially more susceptible to lipid peroxidation than cells in other tissues.

Conversely, monounsaturated fatty acids (MUFAs) provide membrane stability due to their lack of readily oxidizable bis-allylic hydrogen atoms. Stearoyl-CoA desaturase 1 (SCD1), the rate-limiting enzyme in MUFA synthesis, enhances cellular resistance to ferroptosis by modulating the PUFA/MUFA ratio (28). Although direct evidence of SCD1 expression in PDLCs from periodontitis patients is currently lacking, its crucial role suggests that its downregulation may lead to an imbalance in membrane lipid composition and weaken cellular protective capacity. This hypothetical mechanism warrants verification in subsequent molecular biology studies.

#### 2.2.2 Dual-pathway activation of lipid peroxidation

Lethal lipid peroxidation is triggered synergistically by enzymatic and non-enzymatic pathways. In the enzymatic pathway, the lipoxygenase (LOX) family plays a central role: ALOX12 is essential for p53-mediated ferroptosis and serves as a key effector downstream of solute carrier family 7 member 11(SLC7A11) (29); ALOX15 forms a complex with phosphatidylethanolamine-binding protein 1 (PEBP1), specifically catalyzing the peroxidation of PUFAs esterified into phosphatidylethanolamine (30); Cytochrome P450 oxidoreductase (POR) also participates directly in catalysis (31). The non-enzymatic pathway relies on hydroxyl radicals generated by the Fenton reaction to attack PUFA-PLs, initiating a chain reaction of free radicals. Gingival epithelial cells, which highly express ALOX12/15, are highly sensitive to lipid peroxidation-mediated ferroptosis, and their apoptosis exacerbates the breakdown of the gingival barrier (32). PDLCs are highly susceptible to both pathways, and their ferroptosis directly leads to the degradation of the PDLC extracellular matrix.

#### 2.2.3 Coordinated regulation by signaling pathways

In periodontitis, aberrant activation of the Hippo-YAP pathway promotes iron accumulation and lipid peroxidation synergistically by upregulating TFRC and ACSL4 while downregulating GPX4, thereby accelerating ferroptosis-induced periodontal tissue damage (33, 34). Additionally, excessive fatty acid uptake mediated by CD36 can induce ferroptosis in PDLCs (35). The homeostasis of lipid droplets, regulated by factors such as TPD52, PGRMC1, and HIF-2α, also alters the pool of lipid substrates available for peroxidation. The irreversible decomposition of phospholipid hydroperoxides generates highly reactive end-products like 4-hydroxynonenal(4-HNE) and malondialdehyde(MDA) (36). These act as potent electrophiles that covalently modify protein thiol/amino groups, disrupting enzymatic activity and signal transduction. As damage-associated molecular patterns (DAMPs), they activate innate immune receptors to exacerbate inflammation and exhibit genotoxicity. Collectively, these mechanisms drive the loss of tissue homeostasis and sustained damage.

Clinical evidence indicates that gingival tissue biopsies from periodontitis patients show significantly upregulated ACSL4 protein expression, and gingival crevicular fluid exhibits abnormally elevated concentrations of 4-HNE and MDA. Furthermore, ACSL4 expression levels correlate positively with periodontal PD and CAL (6, 37).

### 2.3 Collapse of the antioxidant defense system and pathogenic regulation

Cellular survival relies on a multi-layered antioxidant defense system. Periodontitis systematically disrupts this defensive network through multiple mechanisms, with *P. gingivalis* playing a central regulatory role.

#### 2.3.1 Systemic suppression of the classic antioxidant pathway

The System Xc^--^GSH-GPX4 axis constitutes the core defense pathway against ferroptosis. System Xc^-^ mediates cystine uptake, which is essential for glutathione (GSH) synthesis. GSH serves as the indispensable cofactor for glutathione peroxidase 4 (GPX4), the only selenoprotein capable of directly reducing phospholipid hydroperoxides within membranes. This axis is regulated at multiple levels: microenvironmental stress activates the GCN2-eIF2α-ATF4 pathway to upregulate SLC7A11, while caveolin-1 inhibits ferroptosis by modulating the NOX4/ROS/GPX4 signaling pathway (38, 39). Under simulated diabetic periodontitis conditions, Li et al. identified PDLCs, which highly express SLC7A11 and GPX4, as critical targets of compromised antioxidant defense. *P. gingivalis* lipopolysaccharide (*P. gingivalis* LPS) significantly downregulated both the transcription and protein expression of SLC7A11 via the TLR4/NF-κB pathway, inhibiting cystine uptake, leading to insufficient GSH synthesis and loss of GPX4 activity (40), thereby directly impairing cellular antioxidant capacity.

#### 2.3.2 Comprehensive dysregulation of non-classical pathways

Beyond the classic defense axis, the ferroptosis suppressor protein 1-coenzyme Q10-NAD(P)H (FSP1-CoQ10-NAD(P)H) axis and the GTP cyclohydrolase 1-tetrahydrobiopterin-dihydrofolate reductase (GCH1-BH4-DHFR) axis constitute crucial non-classical ferroptosis-suppressing pathways. FSP1 reduces plasma membrane CoQ10 to ubiquinol to quench lipid radicals, recruits the ESCRT-III complex to repair damaged plasma membranes, and provides an additional scavenging mechanism through vitamin K reduction (41, 42). In periodontitis, excessive ROS downregulates FSP1 expression, compromising its antioxidant function (43). Within mitochondria, dihydroorotate dehydrogenase(DHODH) reduces CoQ10 to provide mitochondrial protection, although its role in periodontal tissues remains to be elucidated. In the GCH1-BH4-DHFR axis, BH4, synthesized by GCH1, acts as a potent lipophilic radical scavenger that selectively protects PUFA-PLs and regulates CoQ10 synthesis, while DHFR maintains BH4 recycling to enhance defense (44, 45). Delineating the mechanistic role of the GCH1-BH4-DHFR axis in periodontitis is critical for a comprehensive understanding of the ferroptosis defense network and the development of targeted therapeutic strategies.

#### 2.3.3 Multidimensional attack by pathogens and mitochondrial damage

Unlike a single acute infection with free bacteria, the subgingival biofilm provides continuous, low-dose, multi-virulence-factor synergistic antigenic stimulation in periodontitis. The pathogenic bacterial complex, centered around *P. gingivalis*, synergistically drives ferroptosis in periodontal cells through multidimensional virulence factors, including lipopolysaccharide, gingipains, and the metabolic end product butyrate (46). This establishes a model within the local periodontal microenvironment, in which bacterial metabolic activity and host immune responses act in concert.

At the immunological level, lipopolysaccharide significantly downregulates SLC7A11 expression by activating the TLR4/NF-κB pathway, thereby limiting cysteine uptake and depleting the glutathione pool (40). At the metabolic level, butyrate accumulation resulting from dysbiosis acts as a histone deacetylase inhibitor, upregulating NCOA4 transcription at the epigenetic level while synergizing with lipopolysaccharide to suppress SLC7A11 expression. Furthermore, pro-inflammatory cytokines upregulate ACSL4 via the STAT3 pathway, promoting the accumulation of polyunsaturated fatty acids (47, 48). Consequently, three pro-ferroptotic pathways coexist within the periodontitis microenvironment: lipopolysaccharide suppresses SLC7A11 expression, butyrate upregulates NCOA4 transcription, and pro-inflammatory cytokines enhance ACSL4 expression. The synergistic action of these three pathways represents a key mechanism underlying the heightened susceptibility of periodontal cells to ferroptosis.

Mitochondria represent another critical target. The LPS-TLR4-JNK/NF-κB axis disrupts mitochondrial membrane potential and cristae architecture while downregulating Parkin expression, thereby blocking PINK1/Parkin-mediated mitochondrial autophagy. This results in the accumulation of damaged mitochondria, thereby establishing a vicious cycle in which mitochondrial damage leads to increased ROS, which in turn promotes ferroptosis. Furthermore, *P. gingivalis* may synergize with other pathogenic symbionts to amplify ferroptosis effects. Gingival epithelial cells, which highly express TLR4 and NCOA4, also become primary targets; their ferroptosis directly triggers extracellular matrix degradation, accelerating the destruction of periodontal supporting tissues.

Clinical studies confirm that the expression levels of SLC7A11 and GPX4 in the gingival tissues of periodontitis patients are significantly lower than in healthy individuals. Gingival crevicular fluid shows significantly reduced GSH concentrations and markedly elevated 4-HNE levels (49). Moreover, GPX4 expression exhibits a significant negative correlation with PD and CAL.

### 2.4 The ferroptosis-inflammation vicious cycle drives chronic periodontitis

Growing evidence indicates that ferroptosis can trigger inflammatory responses, while the inflammatory microenvironment further promotes ferroptosis, forming a self-amplifying vicious cycle that drives the persistent progression of chronic periodontitis. In periodontitis, the sustained operation of this cycle is driven by two key amplifiers: the continuous antigenic stimulation from the subgingival biofilm provides fuel for the cycle, while the hypoxic microenvironment of the periodontal pocket significantly lowers the threshold for its initiation. The following sections will systematically dissect this cycle in terms of its mechanistic basis and pathological consequences.

#### 2.4.1 Mechanism of cycle formation: ferroptosis triggers inflammation, and inflammation exacerbates ferroptosis

The lysis of ferroptotic cells releases DAMPs, such as high-mobility group box 1 (HMGB1), ATP, and mitochondrial DNA, which activate the NOD-LRR-purine domain protein 3(NLRP3) inflammasome in macrophages, promoting the maturation and release of IL-1β and IL-18 (50).

It is noteworthy that ferroptosis and inflammation do not represent a unidirectional causal relationship, but rather form a bidirectional positive feedback loop. The inflammatory microenvironment reshapes cellular ferroptosis sensitivity, significantly lowering the threshold for ferroptosis initiation, thereby further amplifying cellular death. In periodontitis, the sustained stimulation from the subgingival biofilm and the hypoxic microenvironment of the periodontal pocket serve as key amplifiers of this loop. The specific mechanisms include: through multiple virulence factors such as lipopolysaccharide, gingipains, and butyrate, the subgingival biofilm synergistically enhances cellular susceptibility to ferroptosis across three pathways, namely iron metabolism, lipid metabolism, and antioxidant defense, thereby providing continuous fuel for the vicious cycle; dynamic shifts in immune cell subsets, such as the selective death of M2 macrophages, further skewing the microenvironment toward pro-inflammatory states (51). Factors like tumor necrosis factor-α(TNF-α) upregulate NCOA4 and ACSL4 via the NF-κB pathway to mediate ferritin autophagy and PUFA accumulation, while inhibiting glutamate cysteine ligase(GCL) activity and reducing GSH synthesis through the MAPK pathway. This process simultaneously enhances the ferroptosis pathway and weakens antioxidant defenses, directly sensitizing cells (52). The hypoxic microenvironment activates HIF-1α, upregulating transferrin receptor 1(TFR1) and CD36 to enhance iron and fatty acid uptake. In summary, inflammatory factors employ a dual strategy of upregulating pro-apoptotic factors and downregulating protective factors, thereby systematically reducing cellular resistance to ferroptosis. This constitutes a classic sensitization mechanism. This cycle exhibits an amplifying effect in pathological processes, providing a theoretical basis for targeted interventions.

#### 2.4.2 Pathological consequences and clinical significance

This vicious cycle leads to persistent ferroptosis in PDLCs and gingival epithelial cells, triggering periodontal ligament degeneration, disruption of the gingival barrier, and alveolar bone resorption. The resulting tissue damage further promotes the proliferation and enhanced virulence of pathogens such as *P. gingivalis*, forming a self-sustaining “pathogen-ferroptosis-inflammation” closed loop that drives the chronicity and progressive destruction of periodontitis. This review systematically elucidates the core driving role of the ferroptosis-inflammation vicious cycle in the pathogenesis of periodontitis and reveals the pivotal position of immune cells. Notably, its mechanism may vary across periodontitis subtypes (e.g., aggressive vs. chronic forms), requiring validation through larger-scale and stratified analyzes. Targeting critical nodes such as NLRP3, NCOA4, or ACSL4 holds promise for disrupting this cycle, offering novel strategies for precision intervention in periodontitis.

This figure systematically elucidates how *P. gingivalis* synergistically regulates three core pathways via the TLR4/NF-κB signaling pathway: (1) iron metabolism imbalance (upregulation of TFRC and NCOA4 expression leading to intracellular iron overload), (2) lipid metabolism reprogramming (enhanced ACSL4 and LPCAT3 activity promoting polyunsaturated fatty acid phospholipid accumulation), (3) antioxidant defense collapse (downregulation of SLC7A11 and GPX4 triggering glutathione depletion and impaired lipid peroxide clearance). These collectively trigger ferroptosis in periodontal ligament cells and gingival epithelial cells. The damage-associated molecular pattern released by ferroptosis activates the NLRP3 inflammasome in macrophages. Pro-inflammatory factors (e.g., TNF-α) reciprocally enhance ferroptosis susceptibility, forming a self-amplifying vicious cycle that ultimately leads to degradation of periodontal supporting tissues and alveolar bone resorption. Arrows denote activation; T-bars denote inhibition. Key molecular abbreviations are detailed in the main text.

## 3 Mechanisms of ferroptosis-mediated periodontal damage

However, ferroptosis is not a passive endpoint of cellular stress but rather an active pathological driver. Once initiated, ferroptosis-induced lipid peroxidation and iron-dependent oxidative damage exacerbate tissue destruction and accelerate periodontitis progression. Nevertheless, the initiation and progression of ferroptosis in periodontitis are significantly modulated by multidimensional heterogeneous factors, including cell type, individual patient status, disease stage, and microbial composition. Among these, differences in ferroptosis susceptibility across cell types are the most direct, yet their causal roles vary. In some cells, ferroptosis acts as an active driver of tissue destruction, whereas in others, it represents a passive consequence or an accompanying phenomenon of the pathological environment. To systematically elucidate these heterogeneous factors, the following sections will explore four interconnected pathogenic axes that illustrate how ferroptosis cooperatively disrupts the structural integrity and homeostatic function of periodontal tissues. Finally, **Table 1** provides a summary of four dimensions: cell type, patient status, disease stage, and microbial factors.

**Table 1:** Heterogeneity of ferroptosis in periodontitis: a layered overview bridging *in vitro* mechanisms and clinical associations.

Layer	Cell type/Subtype	Core molecular mechanisms	Pathological role	Pathological consequence	Ref
I. *In vitro* mechanism studies					
	Gingival epithelial cells	GPX4/SLC7A11↓, ZO-1/E-cadherin↓	Driver: active inducer of barrier disruption	Barrier disruption, bacterial translocation	(9)
	Periodontal ligament fibroblasts	NCOA4-mediated ferritinophagy, iron overload	Driver: direct executor of soft tissue destruction	Collagen degradation, soft tissue destruction	(50, 51)
	Osteoblasts	GSH depletion, RUNX2/OSX/OCN↓	Driver: direct cause of suppressed bone formation	Suppressed bone formation	(52, 53)
	Osteoclasts	Suppressed NCOA4 autophagy (hypoxia), ferritin accumulation	Accompanied phenomenon: tolerant phenotype, not actively driving	Persistent bone resorption	(54)
	Macrophages (M2 vs. M1)	M2: weak antioxidant defense; M1: pro-inflammatory and tolerant	Passive outcome and Amplification factor: M2 death as outcome, M1 polarization amplifies inflammation	M1/M2 imbalance, inflammation amplification	(55–58)
	Neutrophils	Iron overload, lipid peroxidation, and pathogen stimulation induce ferroptotic features; ferroptosis may promote NETosis via ROS/Fe^2+^	Potential amplification factor	Direct pathological contribution of neutrophil ferroptosis remains unclear	(59–61)
II. Individual patient level					
	Iron overload subgroup	Elevated ferritin in gingival crevicular fluid/serum, TFRC↑	Upstream condition: provides substrate for ferroptosis	Enhanced Fenton reaction increased lipid peroxidation	(64)
	Antioxidant-deficient subgroup	Low baseline GPX4/SLC7A11/GSH	Upstream condition: lowers defense threshold	Vulnerable defense system	(65)
	With comorbid diabetes	Hyperglycemia exacerbates oxidative stress (mechanism inferred)	Upstream condition: aggravating factor	Enhanced ferroptosis-inflammation vicious cycle	(40)
III. Disease stage level					
	Early-stage periodontitis	Mild oxidative stress, localized iron retention, partial GPX4 reduction	Primarily confined to epithelial barrier	Early barrier dysfunction, reversible epithelial damage	(66)
	Advanced periodontitis	Elevated tissue ACSL4, elevated 4-HNE/MDA in GCF	Clinically relevant: correlates with disease severity	Irreversible tissue loss	(67)
IV. Microbe-driven studies					
	*Porphyromonas gingivalis*	LPS/TLR4/NF-κB activation, butyrate/NCOA4 axis	Exogenous trigger: actively induces ferroptosis	Multi-pathway synergistic disruption	(9, 68)

### 3.1 Compromise of the periodontal barrier

The integrity of the gingival epithelium, maintained by intercellular tight and adherens junctions, is a critical first−line physical barrier. Emerging evidence indicates that ferroptosis can compromise this barrier. Shi et al. reported that infection of human immortalized oral epithelial cells (HIOECs) with *P. gingivalis* induced hallmarks of ferroptosis, including reduced GPX4 and SLC7A11 levels alongside upregulated ferritin light chain(FTL). These changes were accompanied by marked downregulation of junctional proteins zonula occludens-1 (ZO-1), E−cadherin, and occludin (9). These *in vitro* findings link ferroptotic signaling to impaired intercellular adhesion and increased epithelial permeability, suggesting a plausible mechanism for enhanced bacterial translocation into deeper periodontal tissues *in vivo*. However, direct clinical evidence demonstrating this causal sequence in patients remains limited and warrants further validation using *in vivo* or ex vivo barrier−function assays.

### 3.2 Destruction of periodontal soft tissues

The integrity and function of PDLFs, essential for soft−tissue homeostasis, are compromised by ferroptosis through a two−fold assault on collagen: synthesis is reduced because PDLF viability and activity are impaired, while degradation is accelerated by ferroptosis−associated ROS and pro−inflammatory mediators (50). Bacterial metabolites such as butyrate further exacerbate PDLF ferroptosis by activating NCOA4−dependent ferritinophagy, which mobilizes intracellular iron and promotes lipid peroxidation, thereby inhibiting PDLF proliferation and collagen production (51). The aforementioned mechanisms, originating from *in vitro* primary cell models, indicate that ferroptosis in periodontal ligament fibroblasts serves as an active driver of soft tissue destruction. *In vivo*, elevated NCOA4 expression has been documented in the gingival tissues of periodontitis patients (13), yet the direct causal link between NCOA4 and collagen degradation awaits further confirmation.

### 3.3 Destruction of periodontal hard tissues

The balance of alveolar bone remodeling is disrupted by ferroptosis, which impairs osteoblastic bone formation while favoring osteoclastic resorption. *In vitro*, *P. gingivalis*−LPS induces ferroptosis in MLO−Y4 osteocytic models, producing GSH depletion, downregulation of osteogenic genes, including runt−related transcription factor 2 (RUNX2), osterix (OSX), and osteocalcin (OCN), and impaired mineralized nodule formation (52, 53). Conversely, osteoclasts in the hypoxic periodontal microenvironment may evade ferroptosis by suppressing NCOA4−mediated ferritinophagy, thereby preserving intracellular ferritin and maintaining resorptive activity (54). These *in vitro* findings reveal potential mechanisms by which ferroptosis regulates bone remodeling, yet the pathological roles of osteoblasts and osteoclasts are fundamentally distinct. Ferroptosis in osteoblasts acts as an active driver, directly suppressing bone formation. In contrast, osteoclasts exhibit a ferroptosis-resistant phenotype under hypoxic conditions, allowing their bone resorption activity to persist. Thus, ferroptosis may tilt bone homeostasis toward net resorption through a dual mechanism: inhibiting bone-forming cells while preserving bone-resorbing cells. However, this hypothesis requires *in vivo* validation using lineage tracing mouse models and *in situ* analysis of patient tissues.

### 3.4 Ferroptosis-driven exacerbation of inflammation

Immune cells play a dual role in periodontitis pathogenesis, acting both as victims of ferroptosis and as amplifiers of downstream inflammation. Yang et al. report that *P. gingivalis* infection induces ferroptosis in macrophages, marked by downregulation of key antioxidant defenses, such as GPX4, FSP1, and nuclear factor erythroid 2-related factor 2 (Nrf2) (43). Loss of these defenses promotes iron accumulation, lipid peroxidation, and consequent reductions in macrophage number and function. Emerging data indicate M2 (anti−inflammatory) macrophages may be more susceptible to ferroptosis under inflammatory conditions, whereas M1 (pro−inflammatory) macrophages appear comparatively resistant (55, 56); this differential sensitivity can perturb the M1/M2 balance toward a pro−inflammatory state. In addition, TNF-α released from ferroptotic cells can further drive macrophage polarization toward an M1 phenotype (57, 58). The above mechanisms are primarily based on *in vitro* induced differentiation models, suggesting that ferroptosis in macrophages exhibits composite characteristics in terms of pathological roles. M2 macrophages, due to their relatively weak antioxidant defenses, undergo ferroptosis first, and the subsequent release of DAMPs and TNF-α further promotes M1 polarization, rendering macrophages a factor in amplifying inflammation. The actual phenotype of this mechanism within the *in vivo* microenvironment of periodontitis patients requires further validation using single-cell sequencing or multiplex immunofluorescence techniques.

In addition to macrophages, neutrophils, as the earliest recruited and most abundant innate immune effector cells in periodontitis, may exhibit interactions between ferroptosis and NETosis that constitute an inflammatory amplification pathway independent of macrophages (59). *In vitro* studies and clinical sample analyzes suggest that iron overload, lipid peroxidation, and persistent pathogenic stimulation in the periodontitis microenvironment can induce ferroptotic features in neutrophils. The lipid peroxidation, ROS burst, and iron release associated with ferroptosis may further promote NETosis, thereby generating a cascade amplification effect that exacerbates periodontal connective tissue degradation and bone resorption (60, 61). It should be noted that direct *in vivo* evidence for neutrophil ferroptosis in periodontitis remains limited, and its pathological contribution is primarily achieved indirectly through NETosis.

Beyond macrophages and neutrophils, other immune cells such as dendritic cells and T and B lymphocytes may also participate in ferroptosis-related immune regulation in periodontitis, collectively forming a multilayered and interconnected immune-ferroptosis network. However, the specific mechanisms underlying these interactions remain to be further explored.

### 3.5 Cascading regulation of multidimensional heterogeneity

The preceding sections have separately elucidated the mechanistic findings of ferroptosis in gingival epithelial cells, periodontal ligament fibroblasts, osteoblasts, osteoclasts, and macrophages. Beyond cell type, the pathological contribution of ferroptosis is subject to cascading regulation by multiple multidimensional factors, including individual patient characteristics, disease stage, and microbial composition. At the cellular level, susceptibility to ferroptosis and the pathological roles of different cell types vary. At the individual patient level, differences in iron overload severity, antioxidant reserves, and systemic comorbidities such as diabetes directly influence the threshold for ferroptosis initiation and its pathological contribution (62). At the disease stage level, ferroptosis is primarily confined to the epithelial barrier in early-stage gingivitis, whereas in advanced periodontitis, it extends to bone tissue and the immune system (9). At the microbial level, heterogeneity in the expression of *P. gingivalis* virulence factors and synergistic interactions within the microbiota may further amplify ferroptotic effects (63). This multidimensional heterogeneity collectively determines the therapeutic response to ferroptosis intervention and represents a key barrier in advancing current research toward precision-targeted strategies.

## 4 Crosstalk of ferroptosis with other programmed cell death modalities

After clarifying the mechanisms and multifaceted destructive effects of ferroptosis in periodontitis, it is important to recognize that this process does not occur in isolation. Notably, different regulated cell death pathways are functionally complementary rather than mutually exclusive during periodontitis progression. Pyroptosis activation is highly dependent on direct stimulation of immune cells by pathogen-associated molecular patterns, and its core pathological effect is the massive release of potent pro-inflammatory cytokines such as IL-1β and IL-18. Therefore, pyroptosis is more prominent during acute exacerbations or active lesions of periodontitis (69). However, the chronic progression phase of periodontitis involves numerous non-infectious metabolic drivers, including iron overload resulting from recurrent bleeding and diminished antioxidant defenses caused by aging or diabetes (70). These damage signals do not directly activate pyroptosis but can efficiently trigger ferroptosis. More critically, the spectrum of cells susceptible to ferroptosis differs fundamentally from that of pyroptosis. Ferroptosis occurs in immune cells such as macrophages and neutrophils, but its most notable feature is the ability to directly induce death in non-immune structural cells, including osteoblasts, periodontal ligament fibroblasts, and gingival epithelial cells. By comparison, pyroptosis occurs predominantly in immune cells (71, 72).

**Figure 2:**
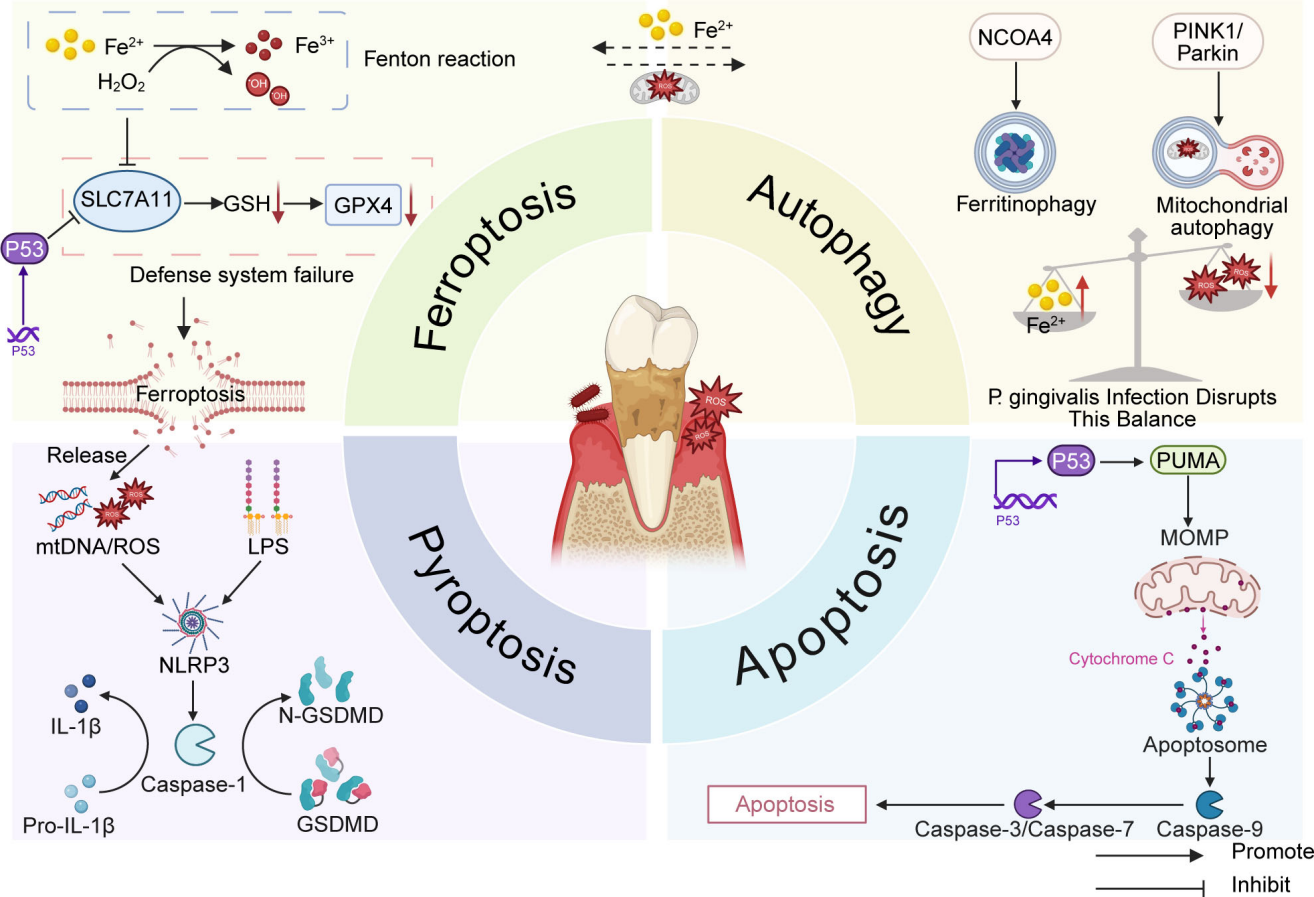
Crosstalk of ferroptosis and other forms of programmed cell death in periodontitis. This figure comprehensively illustrates the interactive mechanisms of ferroptosis, autophagy, pyroptosis, and apoptosis within the periodontal microenvironment, along with their synergistic effects on tissue destruction. The central schematic depicts periodontal tissue architecture, while the four surrounding quadrants illustrate key molecular mechanisms of each programmed cell death pathway. Ferroptosis is driven by lipid peroxidation, relying on Fe²⁺-mediated Fenton reactions, and occurs in close association with dysregulation of the SLC7A11/GSH/GPX4 antioxidant system; p53 promotes ferroptosis by inhibiting SLC7A11. mtDNA and ROS released during ferroptosis activate the NLRP3 inflammasome, triggering caspase-1 activation and GSDMD cleavage. This induces pyroptosis and releases IL-1β and IL-18, forming a ferroptosis-pyroptosis positive feedback amplification loop. Autophagy plays a dual role in this process: PINK1/Parkin-mediated mitochondrial autophagy clears damaged mitochondria to alleviate oxidative stress, exerting an anti-ferroptotic effect; conversely, NCOA4-mediated ferritinophagy releases free iron, exacerbates lipid peroxidation, and promotes ferroptosis. Furthermore, p53 serves as a pivotal regulatory node, inducing apoptosis by upregulating pro-apoptotic factors like PUMA and BAX, while simultaneously promoting ferroptosis by inhibiting SLC7A11. Arrows denote activation; T-bars denote inhibition.

Meanwhile, autophagy exhibits a bidirectional regulatory role in periodontitis, while apoptosis tends to be immunologically silent. Based on these distinctions, this article proposes that pyroptosis acts as an accelerator of inflammatory amplification, whereas ferroptosis serves as an executor of tissue destruction under metabolic stress. These two processes are not mutually substitutive but instead operate at distinct stages of disease progression. The following section will systematically elaborate on the specific interactions between ferroptosis and pyroptosis, autophagy, and apoptosis, as illustrated in **Figure 2**.

### 4.1 A synergistic amplification loop between ferroptosis and pyroptosis

Based on the above conceptual framework, this section further explores the synergistic mechanisms between ferroptosis and pyroptosis in periodontitis, with a focus on how they form a positive feedback amplification loop. The core mechanism underlying the interplay between ferroptosis and pyroptosis is as follows: mitochondrial damage-associated molecular patterns released during ferroptosis, such as mitochondrial DNA and ROS, can be recognized by the NLRP3 inflammasome, leading to caspase-1 activation and subsequent cleavage of gasdermin D to form plasma membrane pores, thereby executing pyroptosis and releasing mature IL-1β and IL-18 (73–76).

This mechanism establishes a self-amplifying positive feedback loop in periodontitis. On one hand, IL-1β, IL-18, and other damage-associated molecular patterns released by pyroptosis recruit and activate additional immune cells (77, 78), further exacerbating oxidative stress and weakening the antioxidant defenses of adjacent periodontal cells, rendering them more susceptible to ferroptosis (37, 79). On the other hand, the sustained occurrence of ferroptosis continuously supplies mitochondrial damage-associated molecular patterns that activate NLRP3, maintaining persistent pyroptosis activation.

This “ferroptosis-pyroptosis-inflammation” closed loop is repeatedly initiated under continuous stimulation by *P. gingivalis* and its lipopolysaccharide, accelerating the loss of functional cells such as periodontal ligament cells and osteoblasts, and promoting soft tissue destruction and alveolar bone resorption (80–82). Notably, in this loop, pyroptosis is primarily responsible for the rapid amplification of acute inflammation, whereas ferroptosis drives disease progression toward an irreversible direction through sustained depletion of structural cells. This distinction suggests that pyroptosis may represent a more prioritized therapeutic target in the early or acute stages of disease, whereas ferroptosis inhibition strategies may hold unique value in the chronic phase or in patients with comorbid conditions such as iron overload or diabetes.

### 4.2 Autophagy in the dysbiosis: a trigger for periodontal ferroptosis

In periodontitis, the dysbiotic microbiota is not a bystander in ferroptosis but actively regulates host autophagy pathways through the secretion of virulence factors. Clinical evidence indicates that the key pathogenic bacterium *P. gingivalis* and its metabolites in the subgingival plaque of periodontitis patients play a central role in regulating the crosstalk between autophagy and ferroptosis (50).

Dysbiosis disrupts autophagic homeostasis through a bidirectional regulatory mechanism. On one hand, oxidative stress and the inflammatory microenvironment induced by pathogenic bacteria activate NCOA4-mediated ferritinophagy. Upregulation of NCOA4 promotes lysosomal degradation of ferritin, leading to a sharp expansion of the intracellular labile iron pool, which provides the essential iron substrate for ferroptosis and directly lowers the threshold for ferroptosis initiation (83). On the other hand, virulence factors of *P. gingivalis* simultaneously inhibit protective mitophagy. Impairment of the PINK1/Parkin pathway results in failure to clear damaged mitochondria in a timely manner, and these dysfunctional mitochondria become a sustained source of ROS, further exacerbating the lipid peroxidation burden (84).

Under healthy conditions, autophagy maintains cellular homeostasis by clearing damaged organelles, exhibiting a protective function (85). Under persistent attack by periodontopathic bacteria, the regulatory direction of autophagy undergoes a fundamental shift: ferritinophagy is activated to release iron, whereas mitophagy is suppressed to accumulate ROS. This bidirectional imbalance in autophagic function transforms an originally protective mechanism into a pro-death signal, creating a synergistic effect with ferroptosis. Therefore, targeting autophagy regulation may represent a potential strategy to interrupt this pathological transformation.

### 4.3 Emerging crosstalk between ferroptosis and apoptosis

Apoptosis plays a complex dual role in periodontitis. On one hand, *P. gingivalis* can induce immune cell apoptosis as an immune evasion strategy, eliminating immune cells to avoid triggering a strong inflammatory response. On the other hand, defective apoptotic cell clearance in chronic periodontitis leads to plasma membrane rupture, shifting the outcome from immune silence to a pro-inflammatory state. In this context, the cross-talk between ferroptosis and apoptosis further influences disease progression.

The tumor suppressor p53 (TP53, best known as p53) serves as a key node integrating both death signals. p53 not only upregulates pro-apoptotic genes such as p53 upregulated modulator of apoptosis (PUMA) and BCL2−associated X protein (BAX) but also promotes ferroptosis by inhibiting SLC7A11 (86–88). In periodontitis, *P. gingivalis*-driven oxidative stress frequently activates p53, positioning it as a decision-maker in cell fate (89). Furthermore, apoptosis requires ATP, whereas ferroptosis can occur even under energy-deprived conditions; in the periodontal microenvironment where ATP may be compromised, cells may preferentially undergo ferroptosis (90). Defective apoptosis, such as reduced caspase activity in chronic periodontitis, can further enhance cellular susceptibility to ferroptosis.

The immunological consequences of these two cell death modalities are fundamentally different: apoptosis is immunologically silent, whereas ferroptosis releases DAMPs such as high mobility group box 1 (HMGB1) and mtDNA, activating the NLRP3 inflammasome and amplifying inflammation (91–93). This shift from silent clearance to inflammatory burst can establish a positive feedback loop of cell loss and persistent inflammation, collectively exacerbating periodontal tissue vulnerability and progressive structural degradation.

This figure comprehensively illustrates the interactive mechanisms of ferroptosis, autophagy, pyroptosis, and apoptosis within the periodontal microenvironment, along with their synergistic effects on tissue destruction. The central schematic depicts periodontal tissue architecture, while the four surrounding quadrants illustrate key molecular mechanisms of each programmed cell death pathway. Ferroptosis is driven by lipid peroxidation, relying on Fe^2+^-mediated Fenton reactions, and occurs in close association with dysregulation of the SLC7A11/GSH/GPX4 antioxidant system; p53 promotes ferroptosis by inhibiting SLC7A11. mtDNA and ROS released during ferroptosis activate the NLRP3 inflammasome, triggering caspase-1 activation and GSDMD cleavage. This induces pyroptosis and releases IL-1β and IL-18, forming a ferroptosis-pyroptosis positive feedback amplification loop. Autophagy plays a dual role in this process: PINK1/Parkin-mediated mitochondrial autophagy clears damaged mitochondria to alleviate oxidative stress, exerting an anti-ferroptotic effect; conversely, NCOA4-mediated ferritinophagy releases free iron, exacerbates lipid peroxidation, and promotes ferroptosis. Furthermore, p53 serves as a pivotal regulatory node, inducing apoptosis by upregulating pro-apoptotic factors like PUMA and BAX, while simultaneously promoting ferroptosis by inhibiting SLC7A11.Arrows denote activation; T-bars denote inhibition.

## 5 Therapeutic perspectives: ferroptosis inhibition in periodontitis

**Figure 3:**
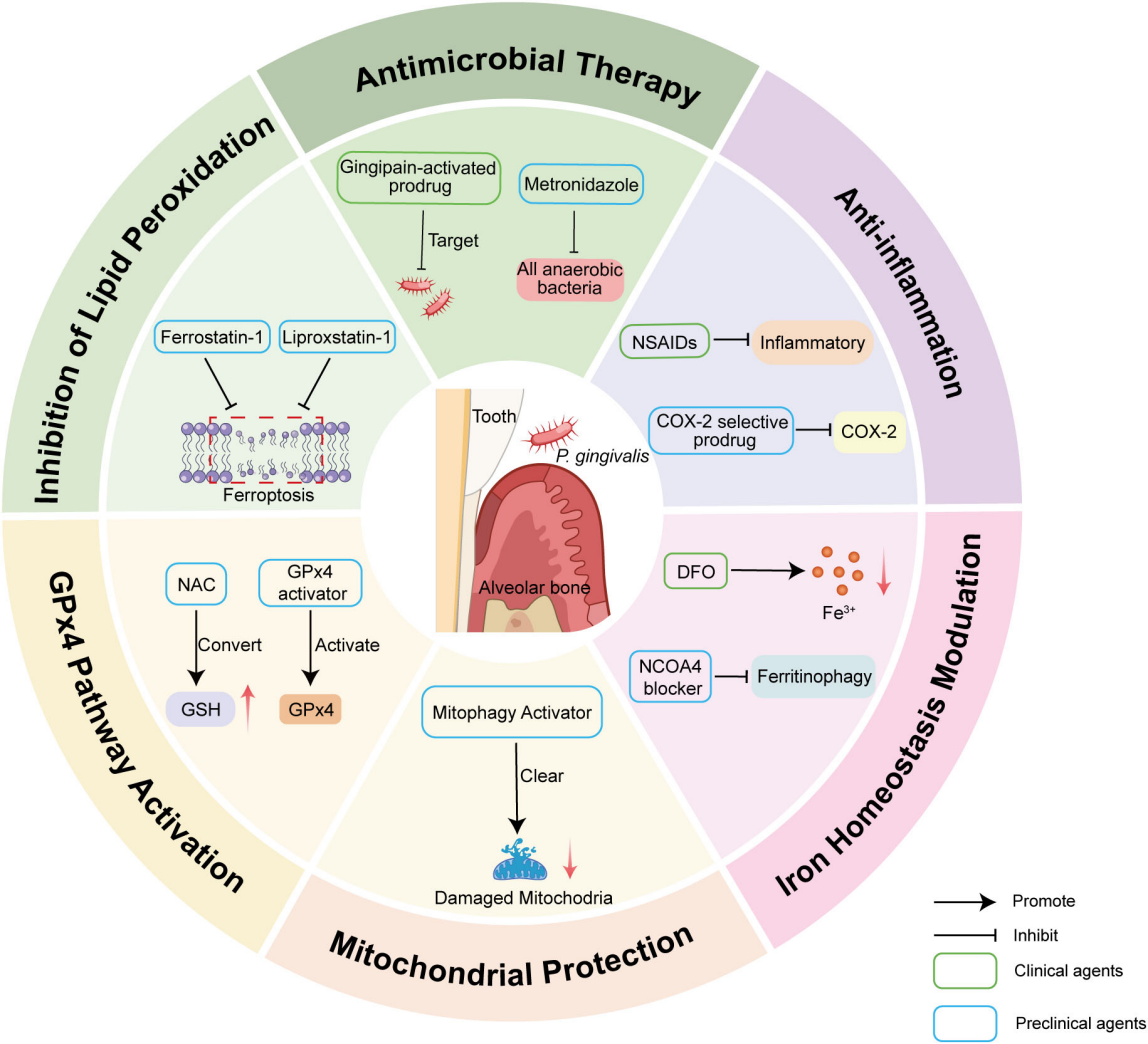
Therapeutic strategies targeting *P. gingivalis*-induced ferroptosis in periodontitis. The central illustration depicts *P. gingivalis* colonization at the gingival margin and adjacent alveolar bone, a setting in which bacterial virulence and host inflammatory responses promote iron accumulation, mitochondrial dysfunction, lipid peroxidation and ultimately ferroptotic cell death. The peripheral sectors summarize six complementary therapeutic strategies: (1) Antimicrobial therapy: agents that directly reduce the anaerobic bacterial load (e.g., metronidazole) or prodrugs activated by bacterial gingipains are shown as approaches to remove the inciting infectious stimulus. (2) Anti‑inflammation: inhibition of inflammatory mediators (e.g., NSAIDs) or selective targeting of COX‑2 via prodrugs is depicted to attenuate inflammation‑driven oxidative stress. (3) Iron homeostasis modulation: iron chelation with deferoxamine (DFO) and inhibition of NCOA4‑dependent ferritinophagy are illustrated as means to lower the labile iron pool and reduce iron‑catalyzed lipid peroxidation. (4) Mitochondrial protection: activation of mitophagy and related pathways is proposed to remove damaged mitochondria, thereby limiting mitochondrial ROS production that fuels ferroptosis. (5) GPX4 pathway activation: provision of cysteine precursors such as N‑acetylcysteine (NAC) to restore intracellular GSH levels, together with direct GPX4 activators, is indicated to enhance enzymatic detoxification of lipid hydroperoxides. (6) Inhibition of lipid peroxidation: small‑molecule ferroptosis inhibitors (e.g., ferrostatin‑1, liproxstatin‑1) are shown to block lipid radical propagation and prevent ferroptotic cell death. (Arrows denote activation; T-bars denote inhibition. Green‑framed labels denote agents with clinical application, blue‑framed labels indicate preclinical agents. The schematic is illustrative and does not imply exhaustive coverage of all potential targets or agents.)

The intricate pathogenic network linking ferroptosis to periodontitis reveals multiple potential nodes for therapeutic intervention, as summarized in **Figure 3**. However, because the ferroptosis−inflammation feed−forward loop can perpetuate tissue damage, conventional anti−infective strategies alone are often insufficient for durable disease resolution. This limitation has stimulated interest in targeted anti−ferroptotic approaches that aim to interrupt this pathological cycle and promote tissue repair. Recent investigative efforts, which act on distinct nodes of the ferroptotic cascade, are summarized in **Table 2** and can be broadly categorized into four principal modalities: ferroptosis inhibitors, natural compounds, genetic modulation, and localized delivery systems. Mechanistically, these strategies function by chelating iron, suppressing lipid peroxidation, enhancing cellular antioxidant capacity, and delivering agents to periodontal sites to limit systemic exposure. Notably, although local delivery strategies theoretically offer site−specific advantages, the periodontal environment imposes two major barriers that significantly limit drug retention time and stability. These include continuous flushing by the gingival crevicular fluid and high concentrations of proteases. Consequently, clinical translation of local drug delivery in periodontitis faces unique challenges that distinguish it from other routes of administration. Translating these concepts into effective therapies will require careful attention to timing, cell−type specificity, combination with anti−infective and anti−inflammatory measures, reliable biomarkers of ferroptotic activity, and rigorous preclinical and clinical validation.

**Table 2:** Summary of anti-ferroptosis interventions for periodontitis management.

Intervention type	Representative agent/molecule	Mechanism of action	Experimental model	Primary outcomes	Limitations	Ref
Ferroptosis inhibitors	Ferrostatin-1 (Fer-1)	Scavenges lipid radicals; inhibits lipid peroxidation and ROS accumulation	Ligature−induced mouse periodontitis; butyrate−treated PDLFs	Restores cell viability; reduces inflammatory cytokines; protects periodontal tissues	Poor *in vivo* stability and pharmacokinetics	(46, 94)
	Deferoxamine mesylate (DFO)	Fe^2+^; inhibits Fenton reaction	Butyrate-treated PDLFs	Reduces lipid peroxidation; improves epithelial barrier function	May inhibit cell proliferation or induce apoptosis at certain doses	(46, 95)
Natural compounds	Resveratrol	Upregulates GPX4 and SLC7A11; restores mitochondrial structure	Ligature-induced mouse periodontitis; MLO-Y4 osteocyte cell line	Alleviates alveolar bone loss; anti-inflammatory and antioxidant effects	Low bioavailability and rapid metabolism	(40, 96)
	Curcumin	Upregulates SLC7A11 and GPX4; downregulates ACSL4 and TFR1	Ligature-induced mouse periodontitis	Increase GSH, reduce MDA, enhance antioxidant defense	Limited target specificity	(97, 98)
Genetic Regulation	lncRNA LINC00616	Silencing leads to miR-370-mediated suppression of TFRC gene expression, reducing cellular iron uptake	*P. gingivalis* LPS-treated PDLSCs	Enhances cell viability; inhibits ferroptosis	Challenges in targeted delivery and clinical translation	(99)
	C3ar or Cth gene	Gene knockout suppresses macrophage polarization to the pro−inflammatory M1 phenotype	A Cth knockout mouse model of experimental periodontitis	Reduces M1 macrophage infiltration and pro−inflammatory cytokines	High risk of off-target effects from ubiquitous gene expression	(100, 101)
Local Delivery Systems	Green Propolis Lipid Nanoparticles	Anti-inflammatory, antibacterial, antioxidant, immunomodulatory, promotes tissue repair	Ovariectomized rats treated with zoledronic acid; ligature-induced mouse periodontitis	Reduces inflammatory cytokines; promotes periodontal repair	Lacks human validation and long-term safety data	(102)
	Gold Nanoparticles (AuNPs)	Inhibits the NF-κB pathway; promotes M2 macrophage polarization	Ligature-induced mouse periodontitis	Improves the inflammatory microenvironment; reduces alveolar bone resorption	Effects on other immune cell types have not been fully investigated	(103)
	Coenzyme Q10 nanomicelles	Protects mitochondrial membrane and mtDNA	Small clinical study: 15 patients with moderate periodontitis	Reduces periodontal oxidative stress markers	Limited clinical sample size; nanocarrier stability and release kinetics need optimization	(104)
	Polydopamine nanoparticles (PDA NPs)	Scavenges ROS; preserves mitochondrial energy metabolism	*P. gingivalis* LPS-induced mouse periodontitis	Attenuates oxidative stress; preserves mitochondrial function	Relationship between particle size and ROS−scavenging capacity unclear	(105)

In summary, the strategies summarized in **Table 2** highlight the potential to limit periodontal tissue destruction by targeting ferroptosis. Most interventions remain at the preclinical stage. Small−molecule ferroptosis inhibitors and natural compounds show efficacy but are constrained by poor pharmacokinetics and bioavailability. Genetic modulation offers high target specificity but faces delivery and safety hurdles. Localized nanodelivery platforms can improve site−specific targeting and reduce systemic exposure, yet their long−term safety, pharmacokinetics, release profiles and therapeutic durability require rigorous validation in relevant large−animal models and clinical trials. For local delivery systems, most current studies rely on static culture or short−term animal models, which fail to adequately recapitulate the complex physiological conditions characterized by the coexistence of gingival crevicular fluid flow and enzymatic degradation. This discrepancy results in a considerable gap between laboratory efficacy and clinical reality. Future research should establish resistance to both fluid flow and enzymatic degradation as core criteria in the design of local delivery systems. Priority should be given to adhesive materials that enable *in situ* retention within the periodontium, or to the development of periodontitis microenvironment−responsive microneedles, in order to achieve precise and durable action at the lesional site. Concurrently, further efforts are needed to improve drug stability and tissue−specific targeting, optimize dosing regimens and release kinetics, identify robust biomarkers of ferroptosis activity to guide therapeutic timing, and explore rational combinations of anti−infective, anti−inflammatory, and anti−ferroptosis strategies. Addressing these translational challenges is essential for advancing ferroptosis−targeted therapy for periodontitis from the laboratory to the clinic.

The central illustration depicts *P. gingivalis* colonization at the gingival margin and adjacent alveolar bone, a setting in which bacterial virulence and host inflammatory responses promote iron accumulation, mitochondrial dysfunction, lipid peroxidation and ultimately ferroptotic cell death. The peripheral sectors summarize six complementary therapeutic strategies: (1) Antimicrobial therapy: agents that directly reduce the anaerobic bacterial load (e.g., metronidazole) or prodrugs activated by bacterial gingipains are shown as approaches to remove the inciting infectious stimulus. (2) Anti−inflammation: inhibition of inflammatory mediators (e.g., NSAIDs) or selective targeting of COX−2 via prodrugs is depicted to attenuate inflammation−driven oxidative stress. (3) Iron homeostasis modulation: iron chelation with deferoxamine (DFO) and inhibition of NCOA4−dependent ferritinophagy are illustrated as means to lower the labile iron pool and reduce iron−catalyzed lipid peroxidation. (4) Mitochondrial protection: activation of mitophagy and related pathways is proposed to remove damaged mitochondria, thereby limiting mitochondrial ROS production that fuels ferroptosis. (5) GPX4 pathway activation: provision of cysteine precursors such as N−acetylcysteine (NAC) to restore intracellular GSH levels, together with direct GPX4 activators, is indicated to enhance enzymatic detoxification of lipid hydroperoxides. (6) Inhibition of lipid peroxidation: small−molecule ferroptosis inhibitors (e.g., ferrostatin−1, liproxstatin−1) are shown to block lipid radical propagation and prevent ferroptotic cell death. (Arrows denote activation; T-bars denote inhibition. Green−framed labels denote agents with clinical application, blue−framed labels indicate preclinical agents. The schematic is illustrative and does not imply exhaustive coverage of all potential targets or agents.).

## 6 Discussion

Synthesizing the foregoing evidence, this article proposes a central hypothesis of ferroptosis in periodontitis: ferroptosis is not merely a passive accompaniment to disease progression, but has the potential to evolve into an important node and signal amplification link within the pathological network. Available evidence suggests that ferroptosis integrates multiple upstream damage signals, including pathogenic bacterial infection, iron overload, and oxidative stress, and may exacerbate tissue destruction and inflammation amplification through positive feedback loops. However, it must be cautiously noted that positioning ferroptosis as a primary driver currently requires rigorous validation and remains inconclusive. While current clinical and basic evidence strongly supports ferroptosis as a key contributing mechanism in periodontitis progression, the extent of its role relative to classical pathogenic factors such as dysbiosis and immune dysregulation, as well as its causal temporality in the natural history of the disease, remains unclear. On this basis, ferroptosis can be regarded as a highly promising therapeutic target, particularly for patient subgroups with iron overload or antioxidant deficiencies. Targeting this pathway may effectively interrupt the aforementioned vicious cycle, although combined anti-infective and anti-inflammatory therapy remains indispensable.

### 6.1 Challenges

However, current research faces several critical challenges. First, existing *in vitro* monoculture systems and short-term animal models fail to recapitulate the chronic progression, polymicrobial ecology, and stage-specific features of human periodontitis, limiting causal ferroptosis studies (106–108). Second, periodontal tissues contain diverse ferroptosis-sensitive cell types, but most studies rely on tissue homogenates or single-cell lines, lacking single-cell resolution and spatial dynamics to identify susceptible subpopulations and their localization (109–111). Third, research remains unbalanced, focusing mainly on macrophages while underexploring ferroptosis interplay with neutrophils, dendritic cells, and lymphocytes, thus hindering a full view of the periodontal immunopathological network (64, 112, 113). Fourth, ferroptosis biomarkers (e.g., MDA, 4-HNE, GPX4 activity) lack standardized protocols and sufficient specificity to distinguish ferroptosis from other oxidative stress or cell death forms, impeding patient stratification and treatment monitoring (114–116). Fifth, rapid gingival crevicular fluid turnover shortens local drug retention, while the periodontitis microenvironment degrades protein/peptide drugs and small-molecule inhibitors, reducing stability and bioavailability (117, 118). Current research has inadequately simulated or addressed these two clinically relevant limitations, and the translational bottleneck of local drug delivery has been largely overlooked. Finally, ferroptosis is not only a pathological executor but also participates in physiological mechanisms such as normal cell turnover and tumor surveillance (119, 120). Thus, extensive blockade of ferroptosis may disrupt iron homeostasis in vital organs including the liver, kidneys, and hematopoietic system (121, 122). This double-edged sword property underscores the necessity of precise temporal control.

### 6.2 Prospect

Future research should further investigate the bidirectional regulation of iron homeostasis. Beyond iron overload, whether iron depletion modulates ferroptosis thresholds by impairing mitochondrial respiratory chain complexes—affecting membrane potential and ROS generation—requires validation using conditional iron transporter knockout, iron chelators, and real-time mitochondrial monitoring (123, 124). On this basis, ferroptosis research in periodontitis should integrate single-cell sequencing with spatial transcriptomics (e.g., Stereo-seq) to construct high-resolution multi-omics maps, combined with clinical parameters (CBCT bone volume, bleeding on probing, clinical attachment loss) to identify susceptible cell subpopulations and map their spatiotemporal networks with osteoblasts, osteoclasts, and immune cells (6, 125–128).

Systematically decipher the functional mechanisms of non-canonical defense pathways (FSP1-coenzyme Q10, GCH1-BH4, and DHODH) in periodontal tissues (129–131), validate their regulatory roles through gain-of-function and loss-of-function experiments, and develop targeted agonists or lipid droplet-directed nanomedicines for therapeutic intervention (132–134). Comprehensive strategies should synergistically inhibit ferroptosis, pyroptosis, and autophagy while integrating mesenchymal stem cell transplantation derived from dental pulp stem cells or induced pluripotent stem cells (135, 136). Synergistic effects and safety should be evaluated in standardized models to concurrently achieve inflammation regulation and tissue regeneration. Smart local delivery systems should respond to ROS bursts, *P. gingivalis* proteases, or acidic pH using multi-signal logic gating for precise lesion-targeted release (137, 138). Biomarker-driven pipelines should include rapid detection of gingival crevicular fluid 4-HNE for patient stratification and monitoring (139).

These approaches will undergo rigorous validation through biomimetic 3D periodontal co-culture models and large animal studies in Beagle dogs (140, 141). In terms of model optimization, biomimetic periodontal flow models or ex vivo periodontal tissue perfusion systems can be established to screen and optimize delivery carriers under conditions that more closely resemble the real clinical environment, thereby narrowing the gap between laboratory research and clinical translation. Collectively, this framework will advance ferroptosis-targeted strategies toward personalized periodontal therapy.

## 7 Conclusion

In conclusion, ferroptosis bridges oxidative stress, iron overload, and lipid peroxidation-induced tissue damage in periodontitis, extending the classical infection–inflammation framework by contributing to host response dysregulation and tissue destruction. Current understanding is limited by simplified *in vitro* and short-term animal models and neglect of the periodontal pocket’s unique environment, which is characterized by fluid flow, protease activity, hypoxia, and low pH, all of which constrain local interventions. Future research should prioritize *in situ*, multi-omics analyzes of ferroptosis and develop standardized salivary or gingival crevicular fluid biomarkers. Therapeutically, targeting ferroptosis with rationally combined inhibitors, stimuli-responsive nanocarriers, and immunomodulators offers a promising precision strategy, provided that delivery systems are designed to tolerate the periodontal pocket environment. Integrating molecular diagnostics with locally responsive systems could transform periodontal care toward restoring host redox homeostasis and tissue stability.
